# The effect of parametric stimulus size variation on individual face discrimination indexed by fast periodic visual stimulation

**DOI:** 10.1186/1471-2202-15-87

**Published:** 2014-07-19

**Authors:** Milena Dzhelyova, Bruno Rossion

**Affiliations:** Psychological Sciences Research Institute and Institute of Neuroscience, University of Louvain, Louvain, Belgium

**Keywords:** EEG, Individual face discrimination, Face perception, Size-invariance, SSVEP

## Abstract

**Background:**

The human brain is frequently exposed to individual faces across a wide range of different apparent sizes, often seen simultaneously (e.g., when facing a crowd). Here we used a sensitive and objective fast periodic visual stimulation approach while recording scalp electroencephalogram (EEG) to test the effect of size variation on neural responses reflecting individual face discrimination.

**Methods:**

EEG was recorded in ten observers presented with the same face identity at a fixed rate (5.88 Hz, frequency F) and different oddball face identities appearing every five faces (F/5, i.e., 1.18 Hz). Stimulus size was either constant (6.5 × 4 degrees of visual angle) or changed randomly at each stimulation cycle, by 2:1 ratio increasing values from 10% to 80% size variation in four conditions. Absolute stimulus size remained constant across conditions.

**Results:**

The base rate 5.88 Hz EEG response increased with image size variation, particularly over the right occipito-temporal cortex. In contrast, size variation decreased the oddball response marking individual face discrimination over the right occipito-temporal cortex. At constant stimulus size, the F/5 change of identity generated an early (about 100 ms) oddball response reflecting individual face discrimination based on image-based cues. This early component disappeared with a relatively small size variation (i.e., 20%), leaving a robust high-level index of individual face discrimination.

**Conclusions:**

Stimulus size variation is an important manipulation to isolate the contribution of high-level visual processes to individual face discrimination. Nevertheless, even for relatively small stimuli, high-level individual face discrimination processes in the right occipito-temporal cortex remain sensitive to stimulus size variation.

**Electronic supplementary material:**

The online version of this article (doi:10.1186/1471-2202-15-87) contains supplementary material, which is available to authorized users.

## Background

The human face is one of the most familiar and salient stimuli in our visual environment. Being able to tell apart individual faces (“individual face discrimination”, i.e. face A *vs.* face B), regardless of their familiarity, is critical for adequate social interactions. Today there is an enormous amount of research carried out to understand the mechanisms of face perception, and a large proportion of studies rely on individual face discrimination measures. Yet, there is still no good understanding of the process of individual face discrimination, and many issues regarding this process remain debated (e.g., which facial cues are important for individual face discrimination, whether this process is primarily holistic/configural or part-based, what its neural basis and developmental course are, etc.).

An important issue concerns the contribution of low-level visual cues to individual face discrimination: to what extent is this process sensitive to changes in low-level properties such as stimulus illumination, contrast, position, or size? In particular, size of a visual stimulus such as a face is an important factor, since human observers are exposed to faces across a wide range of sizes, sometimes simultaneously (e.g., when facing a crowd). Moreover, unlike other low-level manipulations such as variable stimulus positions in the visual field, which trigger eye movements and are associated with attentional and visual lateralization biases, stimulus size is a property that is relatively easy to manipulate and to control in an experimental setting.

Single-cell recordings in the monkey infero-temporal cortex have emphasized size invariance of the selective response to particular shapes, including faces (e.g., [1–4]). In humans, fMRI studies have shown that repetition suppression - the rapid reduction of neural responses caused by the repetition of a given stimulus – for individual faces resists changes of stimulus size in high level visual areas [5–9]. Event-related potential studies have also shown modulation of the occipito-temporal face-sensitive N170 component ([10], for review [11]) as well as the following N250r component [12], following face identity repetition across changes of stimulus size (e.g., [13, 14]). However, to our knowledge, only two studies, using fMRI, have compared individual face repetition effects across size changes (i.e. “same” or “different” faces with and without change of size [6, 9]).

These studies have reported partly inconsistent results. In the first study, repetition suppression effects in high-level face-selective areas did not differ when face stimulus size was constant or varied within a block [6]. Yet, most recently, increased repetition suppression effects were found in the same functional areas for unfamiliar faces presented at a constant size [9]. However, in the latter study [9], 100% size faces were used in the condition with no size variation, while equally large and smaller stimulus sizes were used in the size variation condition. Thus, the effects of absolute stimulus size versus discrimination across size changes could not be dissociated. Most importantly, to date, there is no report of a systematic (i.e. parametric) test of the effect of size variation on the amplitude of neural responses reflecting individual face discrimination, or even high-level visual discrimination in general.

In the present study, our goal was to parametrically test the effect of size variation on neural responses to faces, controlling for absolute stimulus size, and isolating the effect of size variation on the basic response to a face stimulus from its effect on an individual face discrimination response. Faces constitute an ideal type of stimulus to address this issue because the human brain is not only good at categorizing a given stimulus as a face but, as mentioned above, is also especially adept at individualizing exemplars within the category (i.e. individual face discrimination).

A parametric test of the effect of size variation on individual face discrimination requires a sensitive approach, for which the neural response of interest can be identified and quantified objectively. For this reason, we used the approach of fast periodic visual stimulation (FPVS), which results in periodic electrophysiological responses on the human scalp defined as steady-state visual evoked potentials (SSVEPs) [15, 16]. Over recent years, this approach has been developed to identify robust electrophysiological signatures of individual face discrimination objectively (i.e., at a frequency determined by the experimenter) and rapidly (i.e., in a few minutes) ([17–19], for review [20]), with optimal frequency rates peaking at about 6 Hz [19].

**Figure 1:**
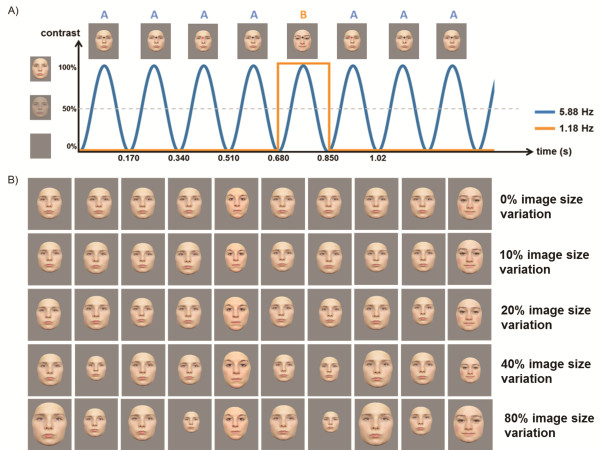
**Experimental design. A)** An illustration of the fast periodic oddball stimulation during EEG recording: a base face **(A)** is presented 4 times followed by a different oddball face **(B)** during each trial, thus there are 2 embedded frequencies: at a rate of 5.88 Hz facial images are presented (base frequency) and at a rate of 1.18 Hz a face with a different identity is shown (oddball frequency). **B)** Varying image size conditions as used in the present study (1st row: 0% image size change, 2nd row: 10% image size change, 3rd row: 20% image size change, 4th row: 40% image size change, 5th row: 80% image size change). Please see also the Additional files 1, 2, 3, 4 and 5 for short movie clips. All variations were implemented in 6 steps, thus the number of possible size changes across the conditions was equal. During each trial the order of size variations was random.

Most recently, Liu-Shuang et al. [21] used FPVS to measure the discrimination of individual faces by presenting a sequence of identical face stimuli at a fast periodic rate (base frequency = F, 5.88 Hz) and introducing different (“oddball”) face stimuli at a slower periodic rate within the sequence (i.e., 1 novel face after 4 identical faces; Figure 1A; see also Figure one in [21]). In this study, a robust individual face discrimination response was recorded over the right occipito-temporal cortex, specifically at the oddball frequency rate (5.88 Hz/5 = 1.18 Hz, corresponding to every 5th novel face) and its harmonics (e.g., 2F/5 = 2.36 Hz, etc.).

This periodic oddball stimulation, with two embedded periodic frequency rates (base rate and oddball rate) carries important advantages for measuring individual face discrimination: within a few minutes of stimulation, it provides high signal-to-noise ratio (SNR) responses in the EEG that are objective, implicit (i.e. without any behavioral task requiring to process the parameter of interest), and easily quantified [20]. Moreover, with this approach, the oddball response is an individual face discrimination response in itself, and thus it can be quantified in each condition without a separately recorded baseline condition in which the exact same face is repeated [21]. Finally, and importantly, there is strong evidence that this individual face discrimination response reflects high-level visual processes: it is largely reduced following face inversion and contrast-reversal, two manipulations that largely preserve low-level visual features but impair behavioral performance at individual face discrimination ([22] for a review on the effect of face inversion, [23] for contrast-reversal effects).

Here, in order to test the effect of size variation on individual face discrimination, we used the paradigm of Liu-Shuang et al. [21] with a parametric size variation manipulation: from 0 to 80% stimulus size variation using a 2:1 ratio scale from 10% (i.e., 0%, 10%, 20%, 40%, 80%; Figure 1B). Critically, absolute size of stimulation was constant across conditions (Figure 1B, please see also the Additional files 1, 2, 3, 4 and 5 for short movie clips.), so that differences in the amplitude of the oddball response across conditions could be entirely associated to interstimulus size variation.

## Results

### Frequency domain analysis

**Figure 2:**
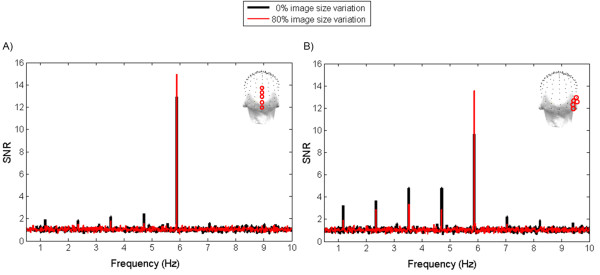
**Grand-averaged SNR spectra (displayed between 0.5 and 10 Hz) over medial occipital (A) and right occipito-temporal (B) regions for the conditions 0 and 80% image size variation.** 3D maps display electrodes position for each ROI. The EEG spectra demonstrate clear responses at the two embedded frequencies: base (F = 5.88 Hz) and oddball frequencies (1F/5 = 1.18 Hz, 2F/5 = 2.36 Hz, 3F/5 = 3.50 Hz, 4F/5 = 4.70 Hz, 6F/5 = 7.05 Hz, etc.). SNR = 1 at noise level.

**Figure 3:**
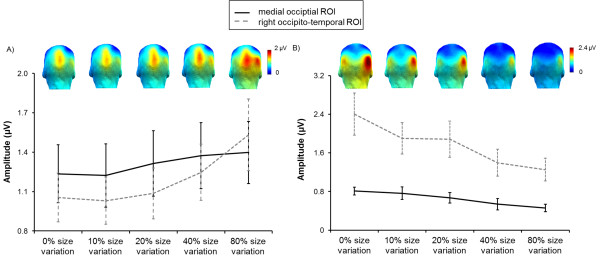
**Mean baseline-corrected amplitudes (±SEM) for the ROIs and topographical maps of the different image size variation conditions: for the base frequency (A) and the summed response (up to 7.05 Hz) for the oddball frequency (B).** The base and the oddball frequency have their own amplitude scales.

Grand-averaged spectra averaged across all electrodes showed clear responses at the 5.88 Hz stimulation frequency (mean baseline-corrected amplitude ± SEM = 0.52 μV ± 0.28; SNR ± SEM = 6.37 ± 0.25), indicating successful synchronization to the visual stimulation. Overall, the response at 5.88 Hz had a medial occipital topography, peaking either on electrode Oz or POOz, depending on the condition (mean baseline corrected amplitude equaled, respectively by electrode, 1.52 and 1.60 μV, SNR equaled 15.77 and 15.91). A similar topography was previously reported when presenting the same face at a base frequency rate [18–21]. With the increase of image size variation, the response increased over the medial occipital region and also spread to the (right) occipito-temporal region(s). Similarly, the activation over the right occipito-temporal region also increased with the increase of image size change variation (see Figures 2A and 3A).

In contrast to the base stimulation frequency, the response to the oddball frequency was larger over the right occipito-temporal region than over the medial occipital region (Figure 2), in line with previous observations [21]. With the increase of image size variation, the oddball response over this region decreased. Similarly, the activation over the medial occipital region also decreased (see Figures 2B and 3B). Values for the baseline-corrected amplitudes, SNR and Z-scores for each condition over the ROIs can be found in Table 1. All response estimations (baseline-corrected amplitudes, SNR and Z-scores) are calculated by taking into account the noise level at the 20 surrounding bins (10 on each side, for details see Methods).

**Table 1:** **Sum of baseline-corrected amplitudes, mean SNR values and corresponding Z-scores (pooled across the ROIs) for the base frequency (A) and for the oddball frequency (B)**

	Amplitude (μV)		SNR		Z-scores	
A) Base frequency						
	rOT	mO	rOT	mO	rOT	mO
0% image size variation	1.06 (0.18)	1.24 (0.22)	9.64 (1.82)	12.94 (1.95)	41.86	76.42
10% image size variation	1.04 (0.18)	1.22 (0.24)	9.24 (1.78)	13.49 (2.31)	40.76	80.39
20% image size variation	1.08 (0.20)	1.32 (0.24)	10.11 (1.95)	14.65 (2.41)	41.31	76.72
40% image size variation	1.24 (0.22)	1.38 (0.26)	10.99 (1.84)	14.06 (2.49)	59.00	99.39
80% image size variation	1.54 (0.28)	1.40 (0.24)	13.55 (1.97)	14.94 (2.14)	62.40	81.66
B) Oddball frequency						
	rOT	mO	rOT	mO	rOT	mO
0% image size variation	2.38 (0.44)	0.74 (0.10)	3.74 (0.51)	1.99 (0.11)	16.47	6.30
10% image size variation	1.84 (0.32)	0.70 (0.14)	3.29 (0.40)	1.93 (0.14)	15.11	5.47
20% image size variation	1.84 (0.38)	0.58 (0.10)	3.18 (0.42)	1.76 (0.13)	13.33	5.72
40% image size variation	1.30 (0.30)	0.44 (0.12)	2.71 (0.42)	1.52 (0.14)	10.49	3.04
80% image size variation	1.22 (0.24)	0.36 (0.08)	2.51 (0.26)	1.46 (0.09)	10.96	2.94

Regions: rOT – right occipito-temporal; mO –medial occipital.

Data were analyzed with repeated measures ANOVA with factors: image *Size variation* (0%, 10%, 20%, 40%, and 80%) and *ROI* (right occipito-temporal and medial occipital) separately for the two frequencies (base stimulation frequency and oddball frequency). If Mauchly’s test of sphericity was significant, a Greenhouse-Geisser correction for degrees of freedom was applied.

### Base frequency

A main effect of *Size variation*, *F*(1.40,12.63) = 5.65, *p* = .025, *η*_*p*_^*2*^ = .39, reflected the 5.88 Hz response increase with the increase of image size variations. Simple contrasts revealed that the response was significantly higher for 80% size variation compared to 0% image size variation, *F*(1,9) = 5.89, *p* = .038, *η*_*p*_^*2*^ = .40; compared to 10% image size change, *F*(1,9) = 7.34, *p* = .024, *η*_*p*_^*2*^ = .45 and compared to 20% image size variations, *F*(1,9) = 5.32, *p* = .046, *η*_*p*_^*2*^ = .37, but did not differ compared to 40% of image size variations, *F* (1,9) = 2.99, *p* = .12, *η*_*p*_^*2*^ = .25.

The main effect of *ROI* did not reach significance, *F*(1,9) = .14, *p* = .72. However, the interaction between *ROI* and *Size variation* was significant, *F*(4,36) = 3.18, *p* = .024, *η*_*p*_^*2*^ = .26, due to a larger increase of the response over the right occipito-temporal region than over the medial occipital region (see also Figure 3A). To further explore this interaction, two ANOVAs with image *Size variation* as a within subject factor for each ROI separately were evaluated. While there was a significant effect of *Size variation* for the right occipito-temporal region, *F*(4,36) = 5.99, *p* = .001, *η*_*p*_^*2*^ = .40, this effect was not significant for the medial occipital region, *F*(4,36) = 2.07, *p* = .11. Moreover, a bootstrap analysis, comparing the slopes of the function predicating the base rate amplitudes across the different image size variations, revealed that the slope was steeper for the right occipito-temporal region (y = .059× + .42) than for the medial occipital region (y = .024× + .58). Indeed, the slope for the right occipito-temporal region fell outside the confidence interval of the medial occipital region [.016; .039].

### Oddball frequency (individual face discrimination)

The ANOVA revealed a main effect of *Size variation*, *F*(4,36) = 12.61, *p* < .0001, *η*_*p*_^*2*^ = .58. Simple contrasts suggested that the amplitude values were significantly smaller for 80% image size variations compared to 0% image size variation, *F*(1,9) = 35.36, *p* < .0001, *η*_*p*_^*2*^ = .80, compared to 10% image size variations, *F*(1,9) = 12.30, *p* = .007, *η*_*p*_^*2*^ = .58 and compared to 20% image size variations, *F*(1,9) = 12.72, *p* = .006, *η*_*p*_^*2*^ = .59. There was also a main effect of *ROI*, *F*(1,9) = 15.40, *p* = .003, *η*_*p*_^*2*^ = .63, indicating a larger response over the right occipito-temporal region (*M* =1.72, *SEM* = .31) than over the medial occipital region (*M* = .54; *SEM* = .06).

These two main effects were further qualified by a significant interaction between *Size variation* and *ROI*, *F*(4,36) = 2.63, *p* = .05, *η*_*p*_^*2*^ = .23, suggesting a larger decrease in amplitude over the right occipito-temporal region than over the medial occipital region. To further explore this interaction, for each ROI, separate ANOVAs with image *Size variation* were evaluated. Although the main effect of *Size variation* was significant for both regions (the right occipito-temporal region, *F*(4,36) = 8.60, *p* < .0001, *η*_*p*_^*2*^ = .49, and the medial occipital region, *F*(4,36) = 4.02, *p* = .009, *η*_*p*_^*2*^ = .31), this effect was stronger for the right occipito-temporal region than for the medial occipital region (see also Figure 3B). Again, a supplementary bootstrap analysis revealed that the slope of the function predicating the oddball frequency amplitudes across the different image size variations was steeper for right occipito-temporal region (y = -.145× + 1.30) than for medial occipital region (y = -.055× + .44). The slope for right occipito-temporal region fell outside the confidence interval of the medial occipital region [-.088; -.040].

### Time domain

**Figure 4:**
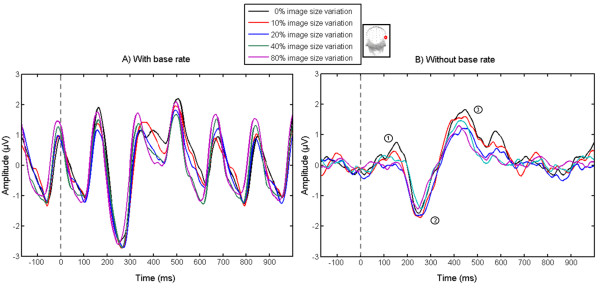
**Grand-averaged EEG waveform on channel P10 during an oddball sequence with a base rate (A) and without (selectively filtered) base rate (B) for the different image size variation conditions.** Time segments start 170 ms before the oddball presentation (marked with a dashed line at 0 ms) and last for 1000 ms after the presentation of the oddball. Three components discriminating the oddball face are revealed.

**Figure 5:**
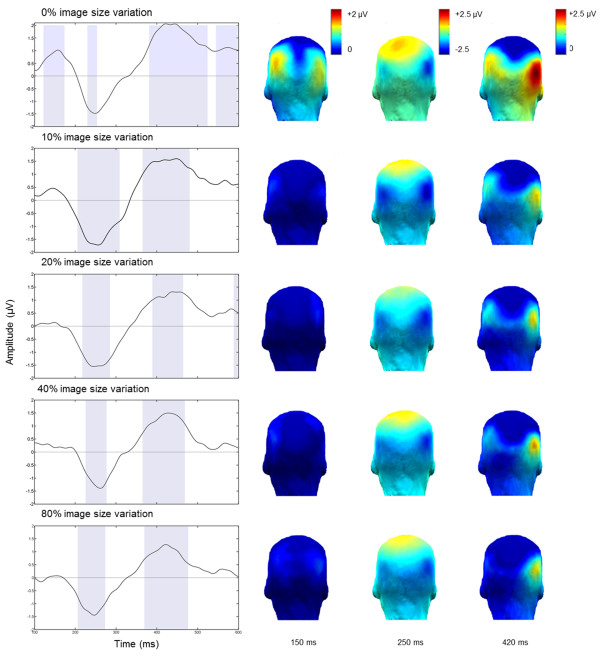
**Components discriminating the oddball from the base face at the occipito-temporal site P10 during one oddball cycle (100 to 600 ms post-stimulus presentation) with significance levels.** Time points when the components are significantly different from 0 (*p* < .05 for 5 consecutive points (250 points/s) at least) are highlighted. Topographical maps represent the regions with maximal response.

The time domain analysis highlighted three components following the change of facial identity (Figures 4 and 5). Importantly, these components cannot be directly related to standard event-related potential (ERP) components because they already reflect the difference between a base face and the oddball faces (i.e. there are differential components). The first component was positive, peaking at about 150 ms post-stimulus. It was significantly different from zero (p < .05 for more than 5 consecutive points, 250 points/s) between 110 and 175 ms post-stimulus only for the condition with no size change and was characterized with a bilateral occipito-temporal topography (Figure 5). Although, this component was present in the 10% condition as well, it was attenuated and did not reach significance based on the defined criterion. The second component, a negativity starting to be significantly different from zero at about 200 ms post-stimulus and reaching its peak at 230-240 ms, was evident for all conditions. This component was distributed over the occipito-temporal region, with clear right hemisphere dominance. In this time window, a positivity over central sites was also apparent. After approximately 400 ms post-stimulus (in the time window 360 – 520 ms, varying across conditions), a third occipito-temporal positive component, with a stronger response over the right hemisphere, was significant in all conditions. All of these components contribute to the robust discrimination response quantified in the frequency-domain as the sum of the oddball harmonics.

## Discussion

Several interesting observations were made regarding the effect of image size manipulation on the periodic electrophysiological. With the increase of the image size variation, we observed an increased response over the regions of interest for the base rate while a reduced response was obtained at the oddball (individual face discrimination) frequency. This dissociation highlights the different processes behind these two responses, as both the base and oddball rate undergo the same image size variations. Furthermore, a complementary time-domain analysis revealed several components manifesting the system’s response when discriminating facial identity.

### The effect of size variation on the base rate periodic response

Overall, changing size at every stimulation cycle does not reduce the basic periodic EEG response, i.e., the base rate SSVEP, which reflects the response generated by the periodic appearance and disappearance of the same face stimulus against a uniform background, roughly 6 times per second. In fact, this response slightly increases over the medial occipital cortex, where the SSVEP is measured in many studies with low-level, and, even more rarely, with high-level, visual stimuli [24–26], and increases relatively more over high-level visual areas (Figure 2).

An increased response with changes of stimulus size likely reflects reduced low-level repetition suppression/adaptation effects. Image size change eliminates strict pixelwise stimulus repetition effects for populations of neurons and, thus, reduces repetition effects due to image-based cues. However, what FPVS measures is the common response to every stimulus in a stimulation sequence. The response of low-level visual areas (e.g., primary visual cortex, V1) is due to the rapid periodic stimulation of the same population of neurons. Thus, when changing stimulus size at every cycle, only a subset of neurons, those coding for the smallest stimulus size (i.e., 2.4 degrees face width as tested in the present study, corresponding roughly to the size of the foveal visual field), are recruited at every stimulation cycle. Populations of neurons coding for perifoveal information, beyond this smallest face size, are recruited at non-periodic rates and should not contribute to the response. Thus, in low-level visual areas the advantage provided by the reduced repetition suppression effect may be counterbalanced by a weaker periodic response when changing stimulus size. Therefore the periodic response in low-level visual areas increases only slightly with increasing size variations.However, in high-level visual areas, populations of neurons have larger receptive fields, and thus are more likely to generate a response at every stimulation cycle, even when the stimulus size changes between stimulation cycles. Thus, responses in high-level visual areas fully benefit from the reduced repetition suppression effects due to the change of size at every stimulation cycle. It follows that a random change of stimulus size at every cycle of FPVS increases the relative contribution of high-level visual areas as compared to low-level visual areas (Figure 3A).

This is an important observation for FPVS studies using high-level visual stimuli. In such studies, the exact same image is usually presented at every cycle [24–26]. Although the findings of these studies are certainly not questioned by our observations, these observations nevertheless suggest that varying a simple factor such as stimulus size (1) is not detrimental for the magnitude of the SSVEP response over low-level visual regions and (2) can increase the relative contribution of high-level visual regions to the recorded response, possibly increasing the sensitivity of the paradigm.

Note that a similar effect of image size change should be expected if other high-level visual stimuli are used. Since the base rate response reflects the response to the visual pattern of stimulation, it does not require a specific expertise at individuating members of the category and, thus, size variations of other visual stimuli would also lead to a decreased response suppression/adaptation. However, these conclusions may only be valid for a specific range of stimulation frequencies. Here we used 6 Hz as a base rate frequency because this rate provides the largest response for faces over occipito-temporal areas [19]. However, at frequency rates of about 10 Hz and beyond, the scalp topography focuses over medial occipital sites and the contribution of high-level areas may be severely limited, so that introducing a size variation between the periodically repeated stimuli may not increase their contribution [19, 20].

### The effect of size variation on individual face discrimination responses

As shown previously [21], the presentation of an oddball face every five faces produced a clear oddball response, indexing individual face discrimination. The exact nature of the mechanisms triggering this oddball response is unclear [20, 21] and could be similar to discrimination response as a result of detection of change [27, 28], predictive coding [29] or release from adaptation [30]. Nevertheless, it is difficult to relate the oddball response obtained with FPVS to transient ERP components elicited during presentation of standard (i.e., base stimuli) and deviant stimuli. For example, the MMN (e.g. [31, 32]) is a negativity evoked when a current stimulus input mismatches a memory representation in the sensory system formed by the preceding stimulus sequence (e.g. [28]). The direct comparison between these two responses is challenging as there are important differences between the paradigms and the two kinds of responses. For instance, while the visual MMN in response to deviant stimuli is reduced in a fixed compared to a randomized oddball sequence [33], a periodic repetition of the oddball is required here in order to identify clear oddball responses in the frequency domain. Moreover, while the negativity of an MMN emerges only following a deviant – standard subtraction, such a subtraction is unnecessary in the present paradigm: the obtained waveform is already a differential response between the base face and the oddball face [21], and contains several differential components spread over time (Figures 4 and 5).

The individual face discrimination response, as reflected in the oddball response to faces, was dominant over the right occipito-temporal cortex. This finding replicates previous observations [21] obtained with a 40% image size variation, and generalizes it to conditions in which the size variation is either absent or negligible, or very large. Despite this replication, size variation had a dramatic impact on the magnitude of the individual face discrimination response over the right occipito-temporal cortex. At these electrode sites, this response was largest when face size did not change, then equally large for 10 and 20% size variation, and then further reduced for 40 and 80% size variations (Figure 3B), an observation which is interesting at two levels at least.

First, the contrast between the effect of size variation on the base rate response and the oddball rate response, at the same right occipito-temporal location, is striking: the exact same manipulation – size variation - *increases* the base rate response while it *decreases* the oddball rate response, showing that these responses truly reflect different neural processes (Figure 3). While the base rate response reflects the contrast between the same face and a uniform background, the oddball rate response reflects the contrast between the frequent face stimulus and the different face identities appearing at every five stimulation cycles. Previously it has been shown that the base rate response can be unaffected by changes such as inversion or contrast-reversal, while the oddball rate response is largely reduced following these manipulations [21]. Here, the dissociation is even more striking, as our size variation manipulation shows that one kind of response can be increased while the other can be simultaneously decreased over the same electrode sites.

Second, there seem to be two steps in the reduction of the oddball response with size variation (Figure 3B). The response reduction from 0% to 10%/20% size variation is the most dramatic because it appears to reflect not only a quantitative decrease but also a qualitative reduction of the response, as suggested by the time-domain analysis and the EEG topographical maps. Indeed, when there is no change in image size, there is a component associated with individual face discrimination, peaking as early as 150 ms following stimulus onset (Figures 4 and 5). Given that the face stimulus is only at full contrast after half a cycle at 5.88 Hz (i.e., 170 ms/2 = 85 ms), this component could probably have an estimated latency of about 100 ms, an extremely early latency for individual face discrimination responses. Although this component is localized over the occipito-temporal cortex, it is bilateral, with no evidence of a right dominance. The absence of this component for the other conditions and the lack of right hemispheric dominance suggest that it reflects the contribution of image-based, or pictorial, cues to individual face discrimination. Note that by pictorial cues, we do not mean exclusively a pixelwise contribution that is independent of the nature of the stimulus, or part of this stimulus, but the recovery from suppression of activity due to the repetition of the exact same face image, which may essentially be a bottom-up process from posterior to anterior visual regions. Similar to these results, in a recent fMRI study [9] a larger repetition suppression effect was found for same size unfamiliar images. Interestingly, this effect disappeared when familiar (famous) faces were presented.

The second and third components identified in the time-domain also look qualitatively different when there is no size variation: compared to the conditions with size variations, the second component is weaker over the right occipito-temporal cortex and increased over medial parietal sites, while the third component is very large but widely distributed over all posterior sites, including medial-occipital sites (Figures 4 and 5). This pattern of observation suggests that in the absence of size variation between the faces presented repeatedly at the base rate, the individual face discrimination response involves low-level visual cues strictly tied to the exact image that is presented, and these cues have an effect all along the time-course of individual face discrimination. To our knowledge, previous EEG studies have not identified such effects [34], which are probably revealed here thanks to the fast periodic oddball paradigm identifying directly, and with a high sensitivity, the differential response between a frequent and a rare stimulus.

However, as soon as a slight variation in stimulation size at the base rate is introduced, the first component disappears, and the subsequent two components vary only quantitatively across the 4 conditions (Figures 4 and 5). This observation suggests that the next two components – and the oddball response quantified in the frequency domain – in these size variation conditions safely index an individual face discrimination process that cannot be attributed to image-based cues, and may also involve recurrent functional processes between face-selective processes at different levels of the ventral visual stream [9]. More specifically, several fMRI studies have found release from repetition suppression to repeated presentations of the same facial identity in two regions of ventral occipitotemporal cortex — the occipital face area (OFA) and the fusiform face area (FFA), and these effects persisted across variations in image size [6, 7, 35, 36].Finally, in the present study, although no qualitative changes could be observed, there was a further reduction of the oddball discrimination response over the right occipito-temporal cortex from 10%/20% to 40%/80% of size variation (Figure 2B). One hypothesis is that this effect is due to limitations in the receptive field of populations of neurons in high-level visual areas, so that some features of the large faces are not perceived, reducing the individual face discrimination response. However, this is unlikely because even the largest faces used in the present study (9.1 × 5.6 degrees in size) were well within the receptive field size of high level visual areas. A more plausible account of this observation is that some of the faces are very small in these conditions, so that facial identity might not be extracted clearly enough to be compared to large size faces, leading to a reduction of the periodic discrimination response. From a practical point of view, this observation suggests that using 10% or maximum 20% of size variation in such paradigms, and with other approaches, may be the best compromise for measuring both high level robust individual face discrimination responses, at least for unfamiliar faces.

## Conclusions

In summary, we made several observations regarding stimulus size variation and its effect on the responses obtained with FPVS. First, random size variation of a repeated face stimulus increases the base rate SSVEP response, particularly over high-level visual regions. This effect could probably be generalized to many kinds of visual stimuli and used to boost the SSVEP and/or the contribution of high-level visual areas to its generation. Second, size variation decreases individual face discrimination over the right occipito-temporal cortex, even for relatively small stimuli that are within the receptive field of high-level visual regions. However, even a substantial size variation (i.e., a factor of 2.33 between the smallest and the largest stimulus size in the 80% condition) does not abolish the individual face discrimination response. Third, without any variation in size, individual face discrimination responses may be contaminated by image-based cues, triggering very early (around 100 ms) visual discrimination responses. Finally, in practice, a 10-20% image size variation is recommended to capture high-level individual face discrimination responses.

## Methods

### Participants

Ten participants (1 male, mean age = 22.28 years, *SD* = 1.45 years, range = 20-25 years) provided signed and informed consent and were paid for their participation in the experiment. They were all right-handed and reported normal or corrected-to-normal vision. None of the participants reported any history of psychiatric or neurological disorder. None reported to have noticed the periodic change of facial identity (1 out of 5 faces). The study was approved by the Biomedical Ethical Committee of the University of Louvain and the study conformed with the 2013 WMA Declaration of Helsinki.

### Stimuli

The stimuli were the same as used in a recent study and will only be briefly described here [21]. They were full-front colored photographs of 25 male and 25 female faces with a neutral expression, placed against a grey background. External features, such as ears and hair, were not visible. Image size of the basic stimuli (i.e., all the faces of the 0% image size change condition) was set to a height of 250 pixels (width = 186 ± 11 pixels) corresponding roughly to 6.53 × 4 degrees of visual angle at a distance of 1 m with a screen resolution of 800 × 600 pixels. Mean luminance of the faces was equalized online during presentation.

### Procedure

The stimuli were presented on a CRT 17-inch (43-cm) monitor controlled by a computer. Participants were seated in a dimly lit room with a 1 m viewing distance to the screen. The whole experiment consisted of 20 trials, containing only 2 trials with female and 2 trials with male faces per condition: 0% (no image size change, all faces at roughly 6.5 × 4 degrees); 10% (varying between 95 and 105% of the image size, i.e. minimum size: 6.2 × 3.8 degrees; maximum size: 6.9 × 4.2 degrees); 20% (varying between 90 and 110% of the image size; i.e. minimum size: 5.9 × 3.6 degrees; maximum size: 7.2 × 4.4 degrees); 40% (varying between 80 and 120% of the image size; i.e. minimum size: 5.2 × 3.2 degrees; maximum size: 7.8 × 4.8 degrees); 80% (60 and 140% of the image size; i.e. minimum size: 3.9 × 2.4 degrees; maximum size: 9.1 × 5.6 degrees), see also Figure 1B and Additional files 1, 2, 3, 4 and 5 for short movie clips. Moreover, all of the size changes were implemented in six levels, so that the number of possible variations was equal across conditions (e.g., the six levels of image size change of 10 % were implemented as the following variations 95%; 97%; 99%; 101%; 103%; 105%; while the six steps of image size change of 40% were 80%; 88%; 96%; 104%; 112%; 120%) and the absolute size of visual stimulation, over a stimulation sequence, did not differ between conditions (Figure 1B).

Each trial started with a fixation cross presented on the screen for a variable duration of 2 to 5 s, followed by 2 s of gradual stimulus fade in, an 80 s stimulation sequence, and 2 s of gradual stimulus fade out. The trial order was randomized across participants. The relatively long duration of the trials guaranteed that the responses of interest are concentrated into discrete frequency bins, thus enhancing their SNR [16, 18]. A custom software running in Matlab was used to display the images at a rate of 5.88 cycles per second (base stimulation frequency) through sinusoidal contrast modulation (see Figure 1A) (e.g. [17, 18]).

Similarly to Liu-Shuang et al.’s study [21], in every trial, one face was randomly selected as the “base face” (A) and repeated throughout the trial. At fixed intervals of every 4 faces, the oddball face, a new identity randomly selected from the remaining 24 faces of the same sex was presented (B, C, D…), resulting in a trial sequence AAAA***B***AAAA***C***AAAA (see Figure 1A). Thus, face identity changes appeared at a frequency of 5.88 Hz/5 = 1.18 Hz. As a result, EEG amplitude at precisely this frequency (F/5 = 1.18 Hz, the oddball frequency) and its harmonics (i.e. 2F/5 = 2.35 Hz, 3F/5 = 3.53 Hz etc.) were used as an index of the visual system’s discrimination of individual faces. Furthermore, as the image size changes were implemented for both base and oddball faces, the response to the oddball face can be attributed to the identity change rather than the change of identity and image size.

Participants were instructed to respond when they noticed a color change of the fixation cross and yet to pay attention to the faces. The fixation cross was presented in the center of the face stimuli, just below the eyes, and briefly (300 ms) changed its color from black to red at random intervals 10 times within every trial. This orthogonal task guaranteed that the participants were attentive. The behavioral data indicated an accurate (M = 96% correct, SD = 2%, range: 86 –100%) and quick (M = 482.52 ms, SD = 55.81 ms) performance of this orthogonal task, without differences across conditions for accuracy, *F*(1, 9) = 0.95, *p* = .45, or correct response times, *F*(1, 9) = 0.40, *p* = .81.

### EEG acquisition

EEG activity was recorded using a BIOSEMI Active-Two amplifier system with 128 Ag/AgCl electrodes. Two additional electrodes (Common Mode Sense [CMS] active electrode and Driven Right Leg [DRL] passive electrode) were used as reference and ground electrodes, respectively. Vertical eye movements were recorded with two electrodes positioned above and below the right eyes. Horizontal eye movements were recorded with electrodes placed at the corner of each eye. EEG and electrooculogram (EOG) recordings were sampled at 512 Hz.

### EEG analysis

#### ROI analyses

We focused our analysis on areas within two sites: the right occipito-temporal region (electrodes PO8; PO10; PO12; P8; P10), where the largest and most consistent responses were observed in previous FPVS studies with faces (e.g., [19, 21]); and the medial occipital region (electrodes Pz, PPOz, POz, POOz, and Oz) where low-level visual responses, SSVEPs in particular, are typically observed and measured (e.g., [21, 24–26]). These two regions also corresponded to the maxima of activity as observed in the present study (see topographical maps in Figure 3).

### Frequency domain analysis

All EEG processing steps were carried out using Letswave 5 (http://nocions.webnode.com/letswave; [37], and Matlab 2012 (The Mathworks). EEG data were 0.10 – 40 Hz band-pass filtered and then downsampled to 250 Hz to save disk space and reduce computational load. The continuously recorded data were cropped in 86-second segments (4 s before and 2 s after each stimulation sequence). Next, noisy or artifact-ridden channels were re-estimated using linear interpolation (not more than 5% of the electrodes; on average across participants, 4 electrodes were interpolated). All data segments were re-referenced to a common average reference. Then, preprocessed data segments were cropped to an integer number of 1.18 Hz cycles, beginning immediately after the fade in until approximately 79.95 seconds (94 cycles, 19986 bins in total). The first two seconds of the fade in were excluded to avoid any contamination by the initial transient responses. Data were averaged in the time domain, separately for each condition (0, 10, 20, 40, and 80% image size variation) and each participant. A discrete Fast Fourier Transform (FFT) was then applied to these averaged segments and amplitude spectra were extracted for all channels.

Two measures were taken: baseline corrected amplitudes and Z-scores for the base stimulation frequency (5.88 Hz) as well as for the oddball frequency (1.18 Hz) and its subsequent harmonics (2F/5, 3F/5, 4F/5, 6F/5, 7F/5). To calculate the response for the oddball frequency, the 5th harmonic, corresponding to the base stimulation frequency, was excluded. Baseline-corrected amplitudes were calculated by subtracting the average amplitude of the 20 surrounding bins (10 on each side, excluding the immediately adjacent bin and the bins containing the highest and lowest amplitudes) from the amplitude at each frequency (e.g., [38]). This procedure is slightly different from our previous studies (e.g., [18]), in which baseline-correction was performed by estimating the signal-to-noise ratio (SNR), i.e., by dividing the signal by the average amplitude at the neighboring frequency bins. Yet, a sum of ratios may not be recommended for quantification of responses by combining a response spread over multiple harmonics. A baseline-subtraction also has the advantage that the amplitude is expressed in microvolt units [39]. Nevertheless, for comparison purposes to previous studies of individual face discrimination with FPVS, SNR values are also reported. The SNR scores were calculated as the ratio of the amplitude at each frequency and the average of the 20 surrounding bins (10 on each side, excluding the immediately adjacent bin and the most extreme high and low bins (e.g., [18, 21]). Grand-averages of the baseline-corrected amplitudes for each condition were calculated. Z-scores were also calculated in a similar way, using the mean and standard deviation of the 20 frequency bins surrounding the frequency of interest.

To define the number of harmonics included in the group data analyses, for each condition, response amplitudes were grand-averaged across participants and pooled across channels to examine the largest harmonic responses (see also [21]). Next, the pooled amplitude data were Z-transformed and Z-scores for the base stimulation rate and for the oddball frequency and its 7 harmonics were extracted. Harmonics were analyzed until they were no longer significant for any of the conditions. Threshold was set to Z > 1.96, p < .025 (one-tailed t-test). According to this criterion, for the oddball frequency, the included harmonics were significant for all conditions until the 6th, at 7.05 Hz. These results were also verified on the spectrum pooled across the ROI.

For the individual data analysis, amplitude spectra of the channels defined in the ROIs were pooled together and baseline-corrected amplitudes were calculated. The sum of the baseline-corrected amplitudes for the oddball frequency and its harmonics defined on the grand-averages (see above) were considered as the system’s individual face discrimination response.

### Time domain analysis

A complementary time domain analysis was performed to visualize the shape of periodic changes time-locked to the oddball stimulus. To remove the dominating response at the base frequency rate, a FFT notch filter with 1 Hz width selectively removed the 5.88 Hz base frequency and its 4 harmonics. Overlapping stimulus locked epochs were segmented starting 1000 ms before the oddball stimulus and lasting for another 1000 ms, an overall duration of 2000 ms. For each trial, 92 epochs were available per participant, per condition (94 oddball images × 850 ms per cycle = 79900 ms, yet for each trial the first and the last epoch were excluded as 1 s before/after the epochs was not available). These epochs were averaged for each participant, baseline-corrected using 100 ms before the onset of the stimulus and then grand-averaged for each condition separately.

## Electronic supplementary material

Additional file 1: **A short example of a stimulation sequence with 0% image size change.** (MOV 8 MB)

Additional file 2: **A short example of a stimulation sequence with 10% image size change.** (MOV 7 MB)

Additional file 3: **A short example of a stimulation sequence with 20% image size change.** (MOV 7 MB)

Additional file 4: **A short example of a stimulation sequence with 40% image size change.** (MOV 7 MB)

Additional file 5: **A short example of a stimulation sequence with 80% image size change.** (MOV 7 MB)

## References

[1] Rolls ET, Baylis GC (1986). Size and contrast have only small effects on the responses to faces of neurons in the cortex of the superior temporal sulcus of the monkey. Exp Brain Res.

[2] Ito M, Tamura H, Fujita I, Tanaka K (1995). Size and position invariance of neuronal responses in monkey inferotemporal cortex. J Neurophysiol.

[3] Schwartz EL, Desimone R, Albright TD, Gross CG (1983). Shape recognition and inferior temporal neurons. Proc Natl Acad Sci.

[4] Lueschow A, Miller EK, Desimone R (1994). Inferior temporal mechanisms for invariant object recognition. Cereb Cortex.

[5] Grill-Spector K, Kushnir T, Edelman S, Avidan G, Itzchak Y, Malach R (1999). Differential processing of objects under various viewing conditions in the human lateral occipital complex. Neuron.

[6] Andrews TJ, Ewbank MP (2004). Distinct representations for facial identity and changeable aspects of faces in the human temporal lobe. NeuroImage.

[7] Cziraki C, Greenlee MW, Kovács G (2010). Neural correlates of high-level adaptation-related aftereffects. J Neurophysiol.

[8] Lee Y, Grady CL, Habak C, Wilson HR, Moscovitch M (2011). Face processing changes in normal aging revealed by fMRI adaptation. J Cogn Neurosci.

[9] Ewbank MP, Henson RN, Rowe JB, Stoyanova RS, Calder AJ (2013). Different neural mechanisms within occipitotemporal cortex underlie repetition suppression across same and different-size faces. Cereb Cortex.

[10] Bentin S, Allison T, Puce A, Perez E, McCarthy G (1996). Electrophysiological studies of face perception in humans. J Cogn Neurosci.

[11] Rossion B, Jacques C, Luck S, Kappenman E (2011). The N170: understanding the time-course of face perception in the human brain. The Oxford Handbook of ERP Components.

[12] Schweinberger SR, Pickering EC, Jentzsch I, Burton AM, Kaufmann JM (2002). Event-related brain potential evidence for a response of inferior temporal cortex to familiar face repetitions. Cogn Brain Res.

[13] Jacques C, d'Arripe O, Rossion B (2007). The time course of the inversion effect during individual face discrimination. J Vis.

[14] Walther C, Schweinberger SR, Kaiser D, Kovács G (2013). Neural correlates of priming and adaptation in familiar face perception. Cortex.

[15] Regan D (1966). Some characteristics of average steady-state and transient responses evoked by modulated light. Electroencephalogr Clin Neurophysiol.

[16] Regan D (1989). Human brain electrophysiology: evoked potentials and evoked magnetic fields in science and medicine.

[17] Rossion B, Boremanse A (2011). Robust sensitivity to facial identity in the right human occipito-temporal cortex as revealed by steady-state visual-evoked potentials. J Vis.

[18] Rossion B, Alonso-Prieto E, Boremanse A, Kuefner D, Van Belle G (2012). A steady-state visualevoked potential approach to individual face perception: Effect of inversion, contrast-reversal and temporal dynamics. NeuroImage.

[19] Alonso-Prieto E, Van Belle G, Liu-Shuang J, Norcia AM, Rossion B (2013). The 6 Hz fundamental stimulation frequency rate for individual face discrimination in the right occipito-temporal cortex. Neuropsychologia.

[20] Rossion B (2014). Understanding individual face discrimination by means of fast periodic visual stimulation. Exp Brain Res.

[21] Liu-Shuang J, Norcia AM, Rossion B (2014). An objective index of individual face discrimination in the right occipito-temporal cortex by means of fast periodic oddball stimulation. Neuropsychologia.

[22] Rossion B (2008). Picture-plane inversion leads to qualitative changes of face perception. Acta Psychol.

[23] Russell R, Sinha P, Biederman I, Nederhouser M (2006). Is pigmentation important for face recognition? Evidence from contrast negation. Perception.

[24] Moratti S, Keil A, Stolarova M (2004). Motivated attention in emotional picture processing is reflected by activity modulation in cortical attention networks. NeuroImage.

[25] Keil A, Moratti S, Sabatinelli D, Bradley MM, Lang PJ (2005). Additive effects of emotional content and spatial selective attention on electrocortical facilitation. Cereb Cortex.

[26] Kaspar K, Hassler U, Martens U, Trujillo-Barreto N, Gruber T (2010). Steady-state visually evoked potential correlates of object recognition. Brain Res.

[27] Kimura M, Schröger E, Czigler I (2011). Visual mismatch negativity and its importance in visual cognitive sciences. NeuroReport.

[28] Czigler I, Balázs L, Winkler I (2002). Memory-based detection of task-irrelevant visual changes. Psychophysiology.

[29] Garrido MI, Kilner JM, Stephan KE, Friston KJ (2009). The mismatch negativity: a review of underlying mechanisms. Clin Neurophysiol.

[30] Grill-Spector K, Malach R (2001). fMR-adaptation: a tool for studying the functional properties of human cortical neurons. Acta Psychol.

[31] Näätänen R, Gaillard AWK, Mäntysalo S (1978). Early selective-attention effect on evoked potential reinterpreted. Acta Psychol.

[32] Pazo-Alvarez P, Cadaveira F, Amenedo E (2003). MMN in the visual modality: a review. Biol Psychol.

[33] Kimura M, Widmann A, Schröger E (2010). Top-down attention affects sequential regularity representation in the human visual system. Int J Psychophysiol.

[34] Eddy MD, Holcomb PJ (2009). Electrophysiological evidence for size invariance in masked picture repetition priming. Brain Cogn.

[35] Gentile F, Rossion B (2014). Temporal frequency tuning of cortical face-sensitive areas for individual face perception. NeuroImage.

[36] Kovács G, Cziraki C, Vidnyánszky Z, Schweinberger SR, Greenlee MW (2008). Position-specific and position-invariant face aftereffects reflect the adaptation of different cortical areas. NeuroImage.

[37] Mouraux A, Iannetti GD (2008). Across-trial averaging of event-related EEG responses and beyond. Magn Reson Imaging.

[38] Nozaradan S, Peretz I, Missal M, Mouraux A (2011). Tagging the neuronal entrainment to beat and meter. J Neurosci.

[39] Hu L, Xiao P, Zhang ZG, Mouraux A, Iannetti GD (2014). Single-trial time–frequency analysis of electrocortical signals: baseline correction and beyond. NeuroImage.

